# Reports on the Hospitals of the United Kingdom

**Published:** 1912-07-06

**Authors:** Henry Burdett


### REPORTS ON THE
### Hospitals of the United Kingdom.

By SIR HENRY BURDETT, K.C.B., K.C.V.O.

### LXVI.—Jessop Hospital for Women, Sheffield.

We inspected this hospital in September 1911, and we found it in the condition described below. Since then the present Chairman of the hospital has spoken to us more than once, and we are glad to hear that the Committee have taken steps to put their house in order. These include, we understand, reorganisation and important changes in the resident staff of the hospital.

This is a depressing hospital to visit, especially when we find that it has more invested funds, upwards of £90,000, than probably any other hospital with only seventy-two beds, of which about half, we understand, are devoted to maternity cases. The atmosphere of the hospital was depressing, for there was evidence everywhere of the absence of a head charged with the duty of overseeing the whole of the staff and of securing the efficient working of the entire establishment and the adequate provision for the comfort of everybody. The wards, and especially the sanitary and other offices in the hospital proper, were on the whole less satisfactory and more slovenly and even dirtier than any hospital we have inspected for years. These faults may in a measure arise from the neglect on the part of the Committee or of those in authority to renew and redecorate the whole hospital and its offices. There seemed to be a lack of discipline amongst the patients and of smartness almost everywhere, with clear evidence of the absence of one controlling and directing head. It is due to the Matron to mention that she was absent on her holiday on September 21, 1911, when we made our inspection. After visiting the older parts of the institution we asked to be shown over the new maternity wards, which we had heard praised, but are not able to say for what reason. Probably the accommodation provided is sufficient, as far as the patients are concerned, but this new unit gave us the impression that it was starved for want of funds, which might account for the really cruel neglect to provide any reasonable comforts for the maternity nurses and midwives, the accommodation in their large recreation room where they have their meals being so poor as to be practically unique in our experience. There was no carpet on the floor, no comfortable chairs, nor anything to make the room either attractive or useable in the restful sense for tired women, or, indeed, for anybody when their working hours are done. The absence of proper bathing and disinfection accommodation for the visiting nursing staff and the miserable slip of a room where they are supposed to keep their outdoor uniform were startling proofs of the absence of a directing and intelligent mind. What this hospital urgently needs is some one capable person who can and will maintain the reputation of this hospital at a high standard. This institution on the whole exhibits the omission on the part of the responsible authorities to discharge their responsibilities adequately by providing ample and up-to-date and comfortable accommodation for the working staff connected with all departments of the hospital. We venture to think, much as this hospital appears to be needed, that until it is thoroughly reconstructed, reorganised, and placed in the hands of some one capable person charged with the duties of making and keeping all the departments efficient, the work which it was founded to do must suffer from many points of view. There is a screw loose somewhere, and the sooner it is discovered and made good the better it will be for the interests of the patients, the comfort of the staff in all departments, and the reputation of the Jessop Hospital for Women, Sheffield.

We publish the above report as it stands, because it forms part of a series and records the actual condition of the hospital at the date to which it relates. Mr. Yeomans was reported to have said at the annual meeting of this hospital that Sir Henry Burdett gave him certain advice, and attributed to him the following: "Carry through all the improvements you can whilst you have the chance, irrespective of the cost, and go to the public afterwards for the money." We take this opportunity to say that either Mr. Yeomans is wrongly reported, or that Mr. Yeomans has attributed to Sir Henry Burdett something which is exactly the opposite to what we have always advocated in regard to voluntary hospital finance. The way to get money for hospitals is to prepare a careful plan of the work to be done and its cost, to ask the public to subscribe the money to enable that work to be done, and to proceed with the work so soon as it has been obtained.

